# Identifying Aortic Arch Branching Variations Using Advanced Imaging Techniques

**DOI:** 10.3390/medicina61112059

**Published:** 2025-11-19

**Authors:** Elisabeth M. Mandler, Moritz Horodecki-Tuchslau, Johannes M. Mittendorfer, Franz Kainberger, Lena Hirtler

**Affiliations:** 1 Center for Anatomy and Cell Biology, Medical University of Vienna, 1090 Vienna, Austria; elisabeth.mandler@meduniwien.ac.at (E.M.M.); hirtlerlab@meduniwien.ac.at (M.H.-T.); johannes.mittendorfer@meduniwien.ac.at (J.M.M.); 2 Social Psychiatric Department, State Clinic Hollabrunn, 2020 Hollabrunn, Austria; 3 Department of Radiology and Image Guided Therapy, Medical University of Vienna, 1090 Vienna, Austria; franz.kainberger@meduniwien.ac.at; 4 Teaching Center, Medical University of Vienna, 1090 Vienna, Austria

**Keywords:** aortic arch, branching pattern, variations, computed tomography angiography

## Abstract

*Background and Objectives*: The branching pattern of the aortic arch (AA) is highly variable, with the typical supra-aortic branching configuration observed in about three out of four cases. Even though some variants carry a heightened risk for certain diseases and intraoperative complications, they are often underrepresented in standard textbooks. One of the earliest meta-analyses on this topic was published by Dr. Herbert Lippert in 1967. This study aims to use modern imaging to identify AA variations, compare the prevalence with Lippert’s findings, and evaluate the relevance of his classification in today’s Central European population. *Materials and Methods*: Computed tomography angiography (CTA) scans of 400 patients were retrospectively analyzed and categorized according to Lippert’s classification of AA variations. The prevalence of each variation was calculated and compared to the results reported by Lippert. *Results*: The typical AA branching was observed in 75.5% of cases. Brachiocephalic trunk variations were the second most common, occurring in 19.5% of patients. Variations involving the left vertebral artery branching directly off the AA had a prevalence of 4%. Additionally, two cases (0.5%) exhibited a thyroidea ima artery originating from the AA, and two cases (0.5%) demonstrated an arteria lusoria with a retroesophageal course. *Conclusions*: Lippert’s classification remains highly relevant in describing supra-aortic branching pattern prevalence within today’s Central European population. Although most variants are of limited clinical relevance, some can become symptomatic or cause complications during medical interventions. Awareness of these variations is therefore essential for optimal patient care.

## 1. Introduction

**Figure 1:**
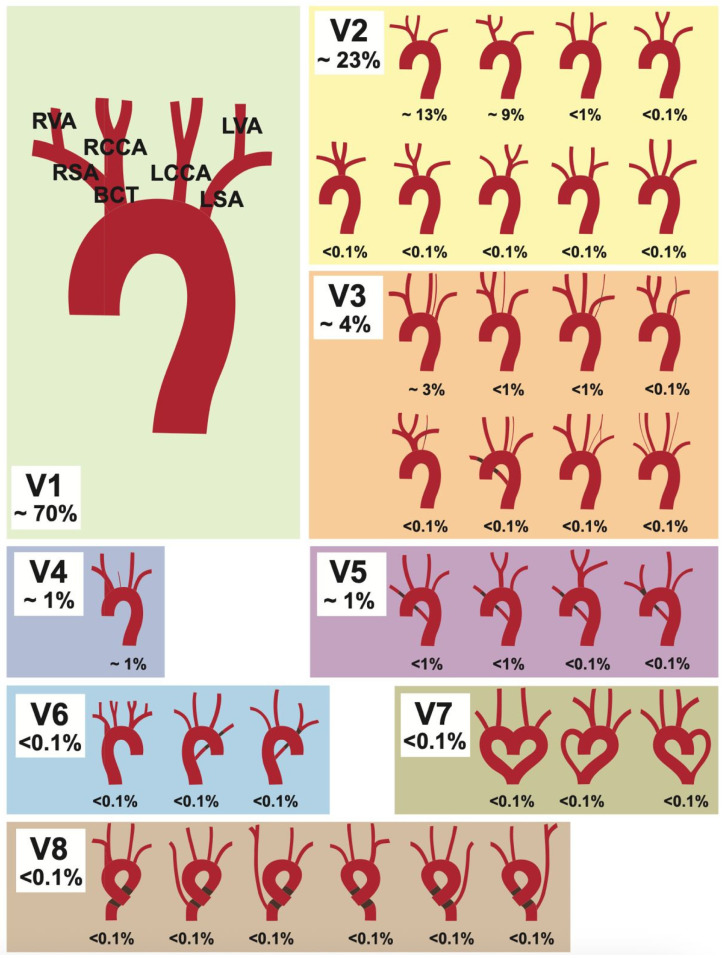
Lippert’s 1967 classification [2] for aortic arch variability. Depicted are the eight variation types (V1–V8), with their subgroups. Figure adapted from [2].

The aortic arch (AA) has a large variety of supra-aortic branch variations due to its complex embryological development [1]. One of the first major reviews on this topic is a 1967 meta-analysis by Herbert Lippert [2], published in the form of supplementary tables in the Journal [Medical Clinic] (German: “Medizinische Klinik”). This original publication was more recently republished with corresponding clinical imaging [3]. In this, Lippert defined eight AA variations, along with numerous subtypes, resulting in over 30 different variations (see Figure 1). Although some variants are relatively common and can cause life-threatening complications during certain procedures [4], they are absent from many standard textbooks. Some studies have shown that specific variants carry a higher risk for certain diseases and intraoperative complications [5,6,7,8]. Examples of this include a higher risk of complications during carotid artery stents in patients with non-typical AA variations [9] and higher rates of thoracic aortic disease [10]. Challenges in patients with acute myocardial infarction are also described, where an aberrant subclavian artery can complicate radial access during coronary intervention [11]. In addition, various AA branching types can cause compression of the trachea or esophagus, leading to symptoms such as dyspnea and dysphagia [12,13,14]. These cases underscore that an understanding of the AA and its branching vessels, including possible anatomical variations, is essential for vascular surgeons and interventional radiologists. This knowledge is crucial for planning and safely executing procedures as well as for accurate interpretation of imaging.

Despite its considerable influence, Lippert’s [2] original work does not exactly specify the investigated population demographics or the specific methods of data collection. Although the more recent edition [3] demonstrates variants using modern imaging, it does not disclose details on the demographics of the patients examined. Therefore, the aim of the present study was to employ modern imaging to identify variations in the aortic arch and supra-aortic vessels in a representative Central European population and evaluate whether Lippert’s extensive meta-analysis still remains relevant or if an update is necessary.

## 2. Materials and Methods

This study was conducted as a retrospective cross-sectional study, carried out at the Division of Anatomy, Center for Anatomy and Cell Biology of the Medical University of Vienna, Austria. All patient data and CT angiographs (CTAs) were obtained from the Department of Radiology and Nuclear Medicine at the General Hospital of Vienna, Austria. Ethical approval was granted by the ethics board of the Medical University of Vienna, Austria (Nr. 1082/2018). Based on a power analysis [15] (presets: confidence level of 95%, margin of error of 5%), a minimum of 385 patients was estimated to achieve adequate statistical power. One CTA was analyzed per patient, with the scan obtained within the six-month time span between 1 July and 31 December 2017. Patients were eligible for inclusion if they were 18 years of age or older. Exclusion criteria included patients with previous vascular surgeries, such as a coronary bypass or stenting of the supra-aortic vessels, in order to avoid distortion of natural variation, as well as CTAs with insufficient resolution or image artifacts in the topographical vicinity of the AA.

The initially included total cohort of patients with a CTA in the predefined time range consisted of 740 patients. After application of all exclusion criteria, the study cohort was composed of 400 subjects—50 male and 150 female individuals with an average age of 65.4 years—whose scans were subsequently evaluated in this study.

The CT angiography scans were acquired using a multidetector CT scanner (Siemens Somatom—Erlangen, Germany). Contrast-enhanced arterial-phase imaging was performed after intravenous administration of a contrast agent with subsequent reconstruction using a standard vascular kernel.

### 2.1. Image Evaluation

All 400 CTAs were analyzed and categorized using Lippert’s 1967 classification [2] for AA variability. This system includes eight variation types (V1–V8), with subgroups indicated by lowercase letters (see Figure 1). The collected data was anonymized and recorded in Microsoft Excel 2013.

Image evaluation was performed in two independent steps. First, all scans were reviewed by the original reviewer (M.H.-T.) for classification according to Lippert’s system. Subsequently, the complete dataset was reviewed again by the senior author (L.H.) to ensure consistency and accuracy. There were no discrepancies between the two reviewers. In cases where vascular anatomy was ambiguous or classification uncertain, all authors jointly reviewed the scans and reached consensus. No conflicts occurred during classification, and no cases were excluded due to unresolved disagreement.

### 2.2. Anatomy of the Aortic Arch and Classification of Variations

The following classification is based on Lippert’s [2] original work, which describes more than 30 supra-aortic variations and will be used for the sake of comparability. Each category and the most common variations are listed and described in Table 1.

The typical AA branching pattern (V1) begins with the brachiocephalic trunk (BCT) as its first branch, which subsequently divides into the right subclavian artery (RSA) and right common carotid artery (RCCA). The RSA gives off the right vertebral artery (RVA). The second and third branches of the AA are the left common carotid artery (LCCA), followed by the left subclavian artery (LSA), with the LSA giving off the left vertebral artery (LVA). The most common brachiocephalic trunk variations (V2) are subgroups V2a (13%) and V2b (3%). Subgroup V2a describes when the BCT and the LCCA share a common trunk. In Subgroup V2b, the LCCA is a branch of the BCT, instead of a direct AA branch. The proper distinction between variants 1, 2a, and 2b can be difficult, as the transition is considered fluid and depends on the research method used [2]. Therefore, differences in percentage distribution are to be expected between different studies. Another common subtype is V3a (3%), in which the LVA is the last branch of the AA. The variant in which the RSA rises directly from the aortic arch (V5) is also referred to as the arteria lusoria (aberrant right subclavian artery). It runs from its origin as the last branch of the AA to the right side of the body, taking a course either behind the esophagus, between the esophagus and the trachea, or in front of the trachea. The circumflex AA (V8) describes a bending of the aortic arch into a left- or right-sided ring before continuing into the descending aorta.

### 2.3. Statistical Analysis

The percentage distributions of variations in each group (V1–V8) and subgroup were calculated using Microsoft Excel (Microsoft 365 Subscription, Microsoft Corporation—Redmond, WA, USA). The results of this study, compared to those of Lippert (1967) [2], are presented in Table 2.

A chi-squared goodness-of-fit test was performed using IBM SPSS Statistics (Version 27, IBM Corporation, Armonk, NY, USA) to compare the observed distribution of aortic arch variations (V1–V8) with the reference frequencies reported by Lippert. Results were regarded as statistically significant at a threshold of *p* < 0.05.

## 3. Results

Table 2 presents the percentage distribution of AA variants identified from the 400 analyzed CTAs, along with the distribution found by Lippert [2].

The chi-squared goodness-of-fit test revealed no significant difference between the two distributions (χ^2^ = 6.99, df = 7, *p* = 0.43).

**Figure 2:**
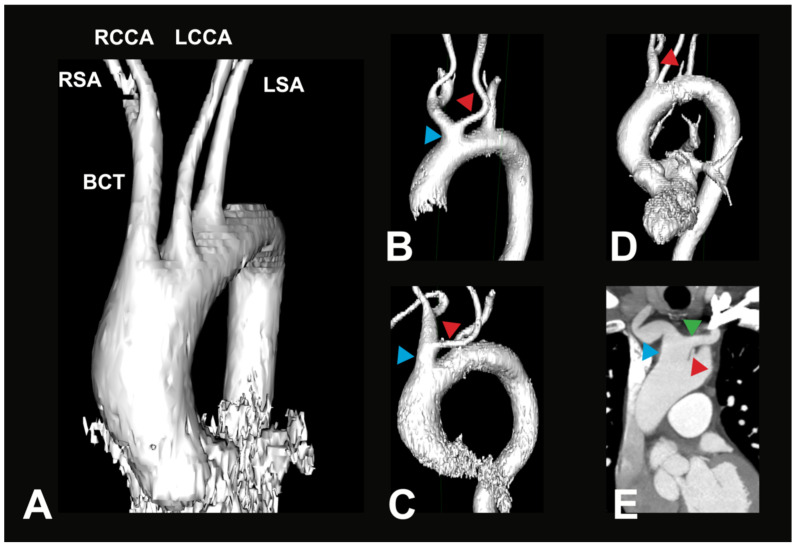
Most common AA variations. (**A**) Exemplary 3D reconstruction of a CTA in a 69-year-old female patient. Depicted is the most common variant of the branches of the aortic arch (V1). BCT—brachiocephalic trunk, RSA—right subclavian artery, RCCA—right common carotid artery, LSA—left subclavian artery, LCCA—left common carotid artery. (**B**) Exemplary 3D reconstruction of a CTA in a 66-year-old female patient. Depicted is a BCT variation (blue arrow), where the LCCA (red arrow) originates off the BCT in the vicinity of the aortic arch (V2a). (**C**) Exemplary 3D reconstruction of a CTA in a 90-year-old female patient. Depicted is a BCT variation (blue arrow), where the LCCA (red arrow) originates from a longer BCT (V2b). (**D**) Exemplary 3D reconstruction of a CTA in a 71-year-old male patient. Depicted is a direct origin of the left vertebral artery (LVA, red arrow) out of the aortic arch; all other arteries originate similarly as in the most common variant (V3a). (**E**) Paracoronal CTA in an 80-year-old male patient. Depicted is a direct origin of the left vertebral artery (LVA, red arrow) out of the aortic arch, in combination with a brachiocephalic trunk variation (blue arrow), where the LCCA (green arrow) originates from the BCT (V3b).

The typical AA branching pattern (V1) shows the highest percentage distribution with 75.5% of all recorded cases in this study (see Figure 2A). BCT variations (V2) form the second largest group, accounting for 19.5% of cases (see Figure 2B). With a significantly smaller, yet not uncommon, prevalence, another variation where the LVA branches directly off the AA (V3) is found in 4% of cases (see Figure 2C). These groups can further be divided into subgroups, which show different percentage distributions between them. The distribution in these subgroups is listed in Table 3A,B.

**Figure 3:**
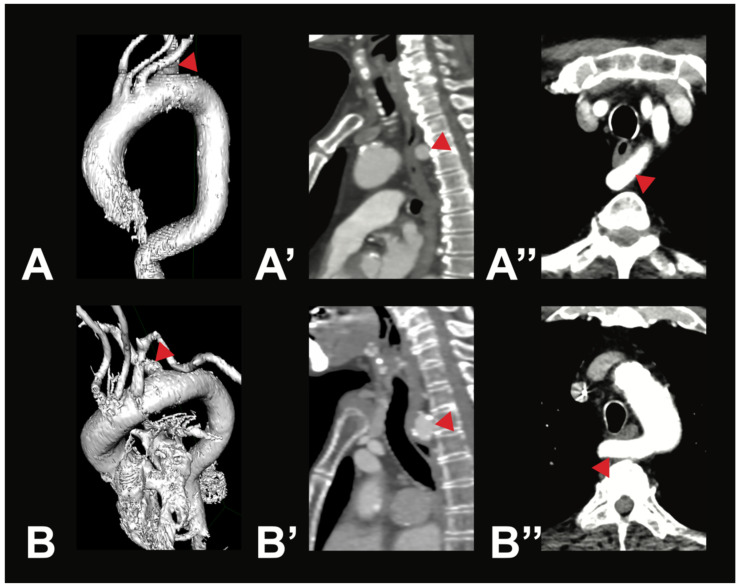
Most symptomatic AA variations—arteria lusoria. Depicted are two exemplary cases of arteria lusoria (V5) in a 71-year-old male patient (**A**—3D reconstruction, **A’**—sagittal view, **A’’**—axial view) and a 79-year-old female patient (**B**—3D reconstruction, **B’**—sagittal view, **B’’**—axial view). In both cases, the RSA (red arrow) was the last branch of the AA, originating from its left side and running posterior to the esophagus to the right side.

Variant V2i was very uncommon, with only one case identified within the examined population. In this variant, the BCT is absent; instead, the RSA and the RCCA arise directly from the AA. Variant V3b was also rare. It is characterized by three AA branches: first, the BCT, which gives rise to the RSA, the LCCA, and the RCCA; second, the LVA; and third, the LSA. Aortic arch branching variations in groups V4–V8 are extremely uncommon, and in some cases, no instances were identified in any of the 400 analyzed CTAs. However, two rare variations were observed in the study population. In two male individuals (0.5%), the thyroidea ima artery was found originating from the AA (V4). Additionally, a variation in which the right subclavian artery arose directly from the AA (V5) was found in two female individuals (0.5%). In both cases, the RSA followed a retroesophageal course (see Figure 3).

## 4. Discussion

An understanding of supra-aortic branch variations and their prevalence within different populations is of significant clinical importance. Certain anatomical variances have been associated with conditions such as congenital heart disease, tracheal compression and dysphagia, as well as thoracic aortic disease [5,16]. One of the first extensive classifications of AA branching variations was provided by Lippert in 1967 [2]. His findings represent a comprehensive overview of not only aortic variations but also arterial variations throughout the human body. Although recently republished in a new edition [3], the therein stated percentual prevalences, especially concerning AA variations, were hitherto never compared to a representative population. The present study was able to verify the continued currency of their results, showing no significant differences between the percentual distribution (see Table 2).

The complexity of arterial variations overall and variations in the AA in particular are well represented in the literature, although most of the existing literature concerning the prevalence of AA variations focuses primarily on left-sided variations. These classifications often categorize variations based on the number of originating branches, limiting this approach as it does not account for all branching patterns. For example, Natsis et al. [17] describes “only” 28 subtypes, whereas Lippert’s classification [2] includes 33 subtypes (for direct comparison, see Table 4).

The most frequent branching pattern of the AA is the normal variant (V1), described with a prevalence of 70% by Lippert [2]. Most available studies report an overall higher percentage in their respective population, including two major review studies. A systematic review of 20 cadaveric studies on AA variations conducted by Natsis et al. [17] showed a prevalence of 78%, while a meta-analysis of 23 imaging studies by Tsiouris et al. [18] showed a prevalence of 77%. Even slightly higher AA variant prevalences were reported by Popieluszko et al. [19] (80.9%) and Murray and Abdel Meguid [1] (78.8%), indicating that our own reported findings (75.5%) are in the middle of the reported range. These minor discrepancies within the literature may be attributed to the fluidity between variants V1, V2a, and 2b, as well as differences in methodology, examiner interpretation, and population demographics [2,17,20].

Apart from the normal variant, the second most common AA variants concern variations in the brachiocephalic trunk (see Table 2). Although the overall prevalence of V2 variations is within the range of other studies, the distribution of subtypes varies between studies. In particular, V2a prevalence ranges from 11% to 16% [2,17,18,19], with our results of 14.7% falling within this range. In contrast, our V2b rate of 4.5% was significantly lower than that reported by Murray [1] (8.66%) and by Tasdemir [21] (24%). Although Tasdemir et al. [21] employed a similar methodology using CTA imaging, the observed differences may be attributed to population-specific factors, as their study was conducted on a Turkish cohort. Interestingly, variant V2a was associated with both an increased incidence of ascending aortic aneurysms and a higher prevalence among embolic stroke patients [7,8]. Syperek et al. [7] suggested that the increased risk for embolic stroke may be related to changed hemodynamic properties within vessels, while the development of aortic aneurysms could be due to differences in vascular tissue thickness between V1 and V2a variants [22]. Only one case in which there was no BCT present (V2i) was found within this study. This is consistent with the very low prevalence in previous studies: Natsis [17] (1%), Tasdemir [21] (0.1%), Lippert [2] (<0.1%), and Tsiouris [18] (0.07%). One variant not observed in our cohort was V2c (bilateral BCT). Both Lippert [2] and Tsiouris [18] reported a prevalence of less than 1%. This absence is possibly a result of our study’s population size, which was determined based on our sample size calculation and thus considered a sufficient cohort size. However, it is also possible that the prevalence of such a variant has nowadays decreased in the Central European population. Others have found a higher prevalence of 3.3% and 2.2% of this variant [1,17].

Variation V3, in which the LVA is a direct branch of the AA, was found with a prevalence of 4% (V3a: 3.5%, V3b: 0.5%) within our examined population. These results are similar to those of Lippert [2] (V3a: 3%, V3b: <1%), Natsis et al. [17] (V3a: 4%, V3b: 1%), Tsiouris et al. [18] (V3a: 3.65%, V3b: 0.1%), and Popieluszko et al. [19] (V3a: 2.3%, V3b: <1%). Murray and Abdel Megiud [1] reported a marginally higher prevalence of V3 variations at 6.2%.

Rarer variations, such as the IMA given off as a direct branch (V4) or the arteria lusoria (V5), were each found in only 0.5% of cases. These values are comparable to previous literature, with V5 reported in under 1% of cases [18,19] and V4 with a prevalence of 0.09% described by Murray [1]. It is important to know that the arteria lusoria can become symptomatic, causing dysphagia lusoria, among other things, through compression of the esophagus [23].

Other variations not observed in our cohort included a right-sided AA (V6), double AA (V7), or circumflex AA (V8). Reported prevalence rates for V6–V8 are scarce in the literature; however, they have been described to occur in less than 0.1% of individuals [2,24]. Zmora et al. [25] discovered a prenatal prevalence of 0.04% for the double AA (V7). The absence of cases reflects not only the rarity of these variations in the general population but also the fact that they are often associated with other fetal abnormalities, which can have poor outcomes, including fetal death [26]. Several AA variations, such as the aberrant RSA, right AA, double AA, and circumflex AA, can form vascular rings surrounding the trachea and esophagus, potentially leading to the compression of these structures [27,28]. This can lead to symptoms like dysphagia, stridor, and dyspnea [16,29]. The overall incidence of vascular rings is estimated at approximately 0.02% [30]. Prenatal diagnosis is possible via ultrasound, which is important since most affected children become symptomatic within the first year of life [31]. Among the various types, double AA is a common form of vascular ring and typically requires surgical repair around the age of 6 months [12]. This AA abnormality can be associated with a high risk of intracardiac abnormalities and a 1% risk of 22q11 microdeletion [32].

In anatomical textbooks and illustrations required during medical training, the most frequent anatomical variant is often depicted. In the case of the AA, this is variant V1, where the arch crosses from right-anterior towards left-posterior, with images first showing the brachiocephalic trunk and then the left common carotid and subclavian arteries. The multitude of possible variations is most often only discovered during further specialization, revealing a multitude of anatomical possibilities. As already shown, anatomical variants, using the example of the arteria lusoria (V4) or other less frequent variations (V6–V8), may be initially responsible for specific symptoms and complaints described by the patients [16,23,29], underlining the importance of topographical knowledge and possible anatomical variants for identifying differential diagnoses. However, even more important is this knowledge when considering increased incidences of pathologies (aneurysms and embolic strokes) in more frequent variants (V2) [7,8], where the unawareness of the responsible clinician of the underlying variant could lead to possible complications in the patient’s treatment. All mentioned variants—of course, depending on the complexity of the variant and the concomitant morphological changes in neighboring structures—therefore warrant a thorough diagnostic and therapeutic approach, including all relevant medical fields, to optimize the patient’s outcome and wellbeing.

### Limitations

Several limitations should be considered when interpreting the results of this study. First, the patients included were exclusively from the General Hospital of Vienna, which limits the diversity of the patient population as well as the total number of cases. However, this was an intentional decision aimed at generating independent findings rather than summarizing previously published data in order to minimize potential bias. Also, a sample size calculation was used to build the basis of the number of patients included in this study; therefore, the results in this study show a sufficiently representative cross-section of the current Central European population. Additionally, this made it possible to directly compare the current finding to those of Lippert [2]. However, to address seldom-occurring AA variations, this study design is insufficient and would have to be adapted to further investigate these rare occurrences, as it was specifically designed to investigate different variation prevalences in the general population.

Second, the included patient population cannot be regarded as healthy, as all individuals presented with a specific indication for their respective CTA. Through thorough definition and restrictive implementation of our exclusion criteria, we tried to minimize a possible selection bias. The presented prevalences of anatomical variants of AA should therefore represent accurate cross-sectional data. Although the resolution of the CTA scans and/or artifacts could influence image interpretation and therefore be a technical limitation, such scans were excluded from the study prior to evaluation.

## 5. Conclusions

In 1967, Prof. Dr. Lippert published a meta-analysis on the subject of arterial variations that is still highly relevant today. It continues to be cited in specialty medical textbooks and was more recently republished in a new edition. Hitherto, the therein stated percentual prevalences, especially concerning AA variations, were never verified. The present study was able to demonstrate that the reported prevalences still remain a valid reference for understanding the prevalence of aortic arch branching patterns.

This continued relevance is important because, although most variants are asymptomatic, awareness and understanding of them are crucial in clinical practice. This knowledge can reduce complications during surgical and interventional procedures, facilitate early detection of pathologies, and improve patient outcomes. Thanks to advances in imaging technology, some of these anomalies can now even be detected prenatally, allowing for early postnatal intervention.

## Figures and Tables

**Table 1:** Variations and subgroups of the aortic arch (AA) variations with percentage distribution described by Lippert (1967) [2]. LVA = left vertebral artery, IMA = thyroidea ima artery, RSA = right subclavian artery.

Variation	Description	Distribution (Lippert)	Subgroups
V1	typical AA branching pattern	70%	
V2	brachiocephalic trunk variations	23%	V2a–i
V3	LVA direct branch of AA	4%	V3a–h
V4	IMA direct branch of AA	1%	
V5	RSA direct branch of AA	1%	V5a–d
V6	right-sided AA	<0.1%	V6a–c
V7	double AA	<0.1%	
V8	circumflex AA	<0.1%	V8a–f

**Table 2:** Percentage distribution (%) of aortic arch variants in the present study compared to Lippert’s [2] results.

Variation	Distribution in This Study	Distribution Found by Lippert
V1: typical AA branching pattern	75.5%	70%
V2: brachiocephalic trunk variations	19.5%	23%
V3: LVA direct branch of AA	4%	4%
V4: IMA direct branch of AA	0.5%	1%
V5: RSA direct branch of AA	0.5%	1%
V6: right-sided AA	0%	<0.1%
V7: double AA	0%	<0.1%
V8: circumflex AA	0%	<0.1%

**Table 3:** (A) Percentage distribution of variant V2: brachiocephalic trunk variations; (B) percentage distribution of variant V3: left vertebral artery as a direct branch of the aortic arch.

(A)		(B)	
V2: Brachiocephalic Trunk Variations	Distribution in This Study	V3: LVA Direct Branch of AA	Distribution in This Study
V2a	14.7%	V3a	3.5%
V2b	4.5%	V3b	0.5%
V2c–h	0%	V3c–h	0%
V2i	0.3%		

**Table 4:** Correspondence between Lippert’s classification and the classification using branch numbers.

Lippert Classification [2]	Branch Number Classification	Branching Pattern
V1	3b1	BCT-LCCA-LSA
V2a	2b1 + 2b2	CT(BCT-LCCA)-LSA
V2b	2b1*	BCT(RSA-RCCA-LCCA)-LVA-LSA
V2c	2b3	BCT-CT(LCCA-LSA)
V2i	4b5	RSA-RCCA-LCCA-LSA
V3a	4b1	BCT-LCCA-LVA-LSA
V3b	3b2	CT(BCT-LCCA)-LVA-LSA
V4	4b3	BCT-IMA-LCCA-LSA
V5a	4b2	RCCA-LCCA-LSA-RSA

## Data Availability

The data presented in this study are available on request from the corresponding author.

## Abbreviations

AA	aortic arch
BCT	brachiocephalic trunk
RSCA	right subclavian artery
LSA	left subclavian artery
RCCA	right common carotid artery
LCCA	left common carotid artery
RVA	right vertebral artery
LVA	left vertebral artery
IMA	thyroidea ima artery
